# A Survey of Insulin-Dependent Diabetes—Part I: Therapies and Devices

**DOI:** 10.1155/2008/405796

**Published:** 2008-07-06

**Authors:** Daisuke Takahashi, Yang Xiao, Fei Hu, Michael Lewis

**Affiliations:** ^1^Department of Computer Science, The University of Alabama, Tuscaloosa, AL 35487-0290, USA; ^2^Department of Electrical and Computer Engineering, The University of Alabama, Tuscaloosa, AL 35487-0286, USA; ^3^Department of Computer Engineering, Rochester Institute of Technology, Rochester, NY 14623-5603, USA

Our survey paper [1] contains a couple of reference mistakes, which should be corrected as follows.

- In [1, page 7], “Table 1: Specification comparison of commercial insulin pumps” should be changed to “Table 1: Specification comparison of commercial insulin pumps, based on [50].”
- In [1, page 10], “Table 2: Specification comparison of commercial blood glucose sensors” should be changed to “Table 2: Specification comparison of commercial blood glucose sensors, based on [51].”
- In [1, page 15], two more references should be added:
  1. [50] http://www.diabetesnet.com/diabetes_technology/ insulin_pump_models.php;
  2. [51] http://www.childrenwithdiabetes.com/continuous.htm.
